# Supplementary material for: Formation of False Context Fear Memory Is Regulated by Hypothalamic Corticotropin-Releasing Factor in Mice
Source: Int J Mol Sci. 2022 Jun 3;23(11):6286. doi: 10.3390/ijms23116286 (PMC9181353; doi:10.3390/ijms23116286)

Revised version of Manuscript ID: ijms-1718457 for *Int. J. Mol. Sci.* (version 5.22.2022)

Title: Formation of false context fear memory is regulated by hypothalamic corticotropin-releasing factor in mice

Authors: Emi Kasama[1], Miho Moriya[1], Ryuma Kamimura[1], Tohru Matsuki[2]*, Kenjiro Seki[1]*

Affiliations: [1]Department of Pharmacology, School of Pharmaceutical Science, Ohu University, 31-1 Misumido, Tomitamachi, Koriyama, Fukushima 963-8611, Japan

[2] Department of Cellular Pathology, Institute for Developmental Research, Aichi Developmental Disability Center, Kasugai, Aichi 480-0392, Japan

***Corresponding authors:**

Kenjiro Seki, Ph.D., 31-1 Misumido, Tomitamachi, Koriyama, Fukushima 963-8611, Japan

Tel.: +81-24-932-9179, E-mail: k-seki@pha.ohu-u.ac.jp

Tohru Matsuki, Ph.D., Kasugai, Aichi 480-0392, Japan

Tel.: +81-568-88-0811, E-mail: matsukit@inst-hsc.jp

Supplementary Materials

Methods and Materials

Mice were placed in Box A for 5 min with CS (group A-A CS (+)) or without CS (group A-A CS (-)). Then, 24 h later, the mice were re-exposed to Box A for 2.5 min. Mice were placed in Box A for 5 min with CS (group ABA CS (+)) or without CS (group ABA CS (-)), and 3 h later, the mice were exposed to Box B for 2.5 min. After an additional 24 h, the ABA CS (+) and ABA CS (-) groups of mice were re-exposed to Box A for 2.5 min. Minimum freezing detection using ANY-maze software was 1 s in each box. The central zone was defined as the middle area (172.5 × 172.5 mm) of the arena in Box B. The distance traveled (m) in the central zone was recorded using the ANY-maze software.

Statistical analysis

Statistical analyses were performed using the EZR (Easy R) software [1] (version 1.38; Saitama, Japan) for the two-way repeated measures ANOVA test. The BellCurve software for Excel (version 3.20). [2] was used as appropriate. Welch's *t*-test was used for **Suppl. Fig. S1C**. Two-way repeated measures ANOVA, followed by Tukey's multiple comparison test and the paired *t*-test between 3 and 24 h in Box B, was performed for **Fig. S1D**. All data presented as bars indicate the mean ± standard error of the mean (SEM). All analyses were set at $p < 0.01$ (**) or $p < 0.05$ (*).

Supplementary figure legends

Figure S1. (**A**) Experimental paradigms for contextual fear memory for A-B CS (-), A-B CS (+), ABB CS (-), and ABB CS (+). Electric shocks (1.0 mA for 2 s × 3 times at 100-s intervals) were delivered to the mice for CS (+);. (**B**) Percentage (%) of freezing time during Context A when

the electric shocks (CS (+)) were delivered at 100 s after the mice were placed in the box. Arrows represent electric shock delivery at 180, 280, and 380 s after the mouse was put in the box. Freezing level was recorded every 1 min. (**C**) Bar graphs showing the percentage of freezing time in Box A 24 h in A-A CS (-) (n = 5) and A-B CS (+) (n = 6) mice; (**D**) Bar graphs showing the percentage of freezing time in Box B at 3 h and Box A at 24 h in ABA CS (-)(n = 5) and in ABA CS (+) (n = 14)mice; (**E**) Percentage (%) of freezing level differences between 3 and 24 h in ABB CS (-) (n = 5), ABB CS (+) (n = 14), ABA CS (-) (n = 5), and ABA CS (+) (n = 14) mice. Data are represented by mean ± SEM. NS represents no significant difference. * $p < 0.05$; ** $p < 0.01$.

Figure S2. Construction and characterization of adeno-associated virus (AAV) PHP.eB vectors for knockdown of mouse *Crf* by shRNA system and overexpression of mouse CRF. (**A**) Illustration of the AAV vectors expressing Crf shRNAs under the control of U6 promoter. The vectors express GFP as a reporter gene under the control of Chicken β-Actin promoter (CBA pro). (**B**) Illustration of the AAV vector carrying CRF*-FLAG-T2A-RFP. CRF* represents the shRNA#2 resistant version. (**C**) Western blotting for expression of CRF*-FLAG-T2A-RFP carrying AAV plasmid that was transfected to HEK293 cells; (**D**) The knockdown validation of the effective shRNA candidates. #2 shRNA was the most effective, and so was used in further experiments.

Figure S3. Original blots showing the detection of FLAG (CRF) and β-actin (full membrane corresponding to cropped blots in Fig. 3C and D indicated by the red dashed line).

References

1. Kanda, Y. Investigation of the freely available easy-to-use software 'EZR' for medical

statistics. *Bone Marrow Transplant* **2013**, *48*, 452-458, doi:10.1038/bmt.2012.244.

2. Kimura, K.I.; Minami, R.; Yamahama, Y.; Hariyama, T.; Hosoda, N. Framework with cytoskeletal actin filaments forming insect footpad hairs inspires biomimetic adhesive device design. *Commun Biol* **2020**, *3*, 272, doi:10.1038/s42003-020-0995-0.

Kasama et al, Supplementally Figure S1

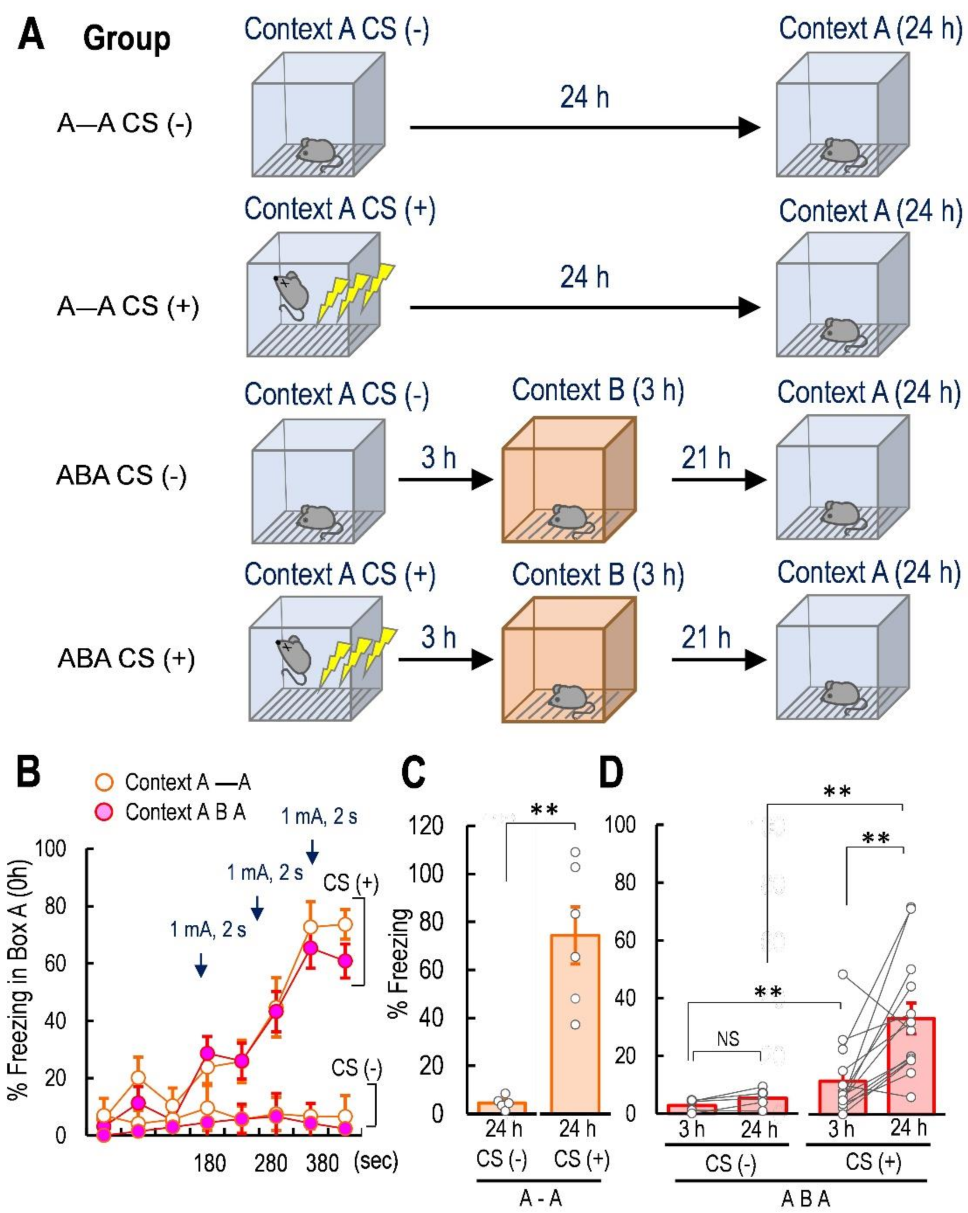

Kasama et al, Supplementally Figure S2

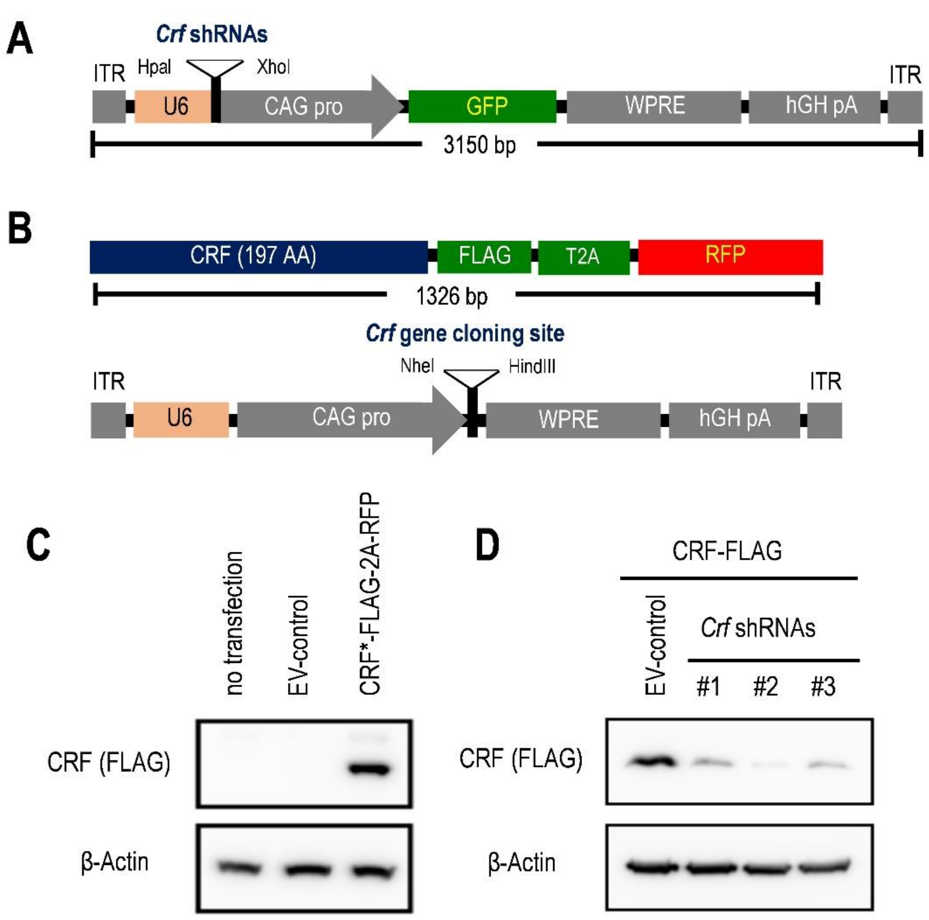

Kasama et al, Supplementally Figure S3

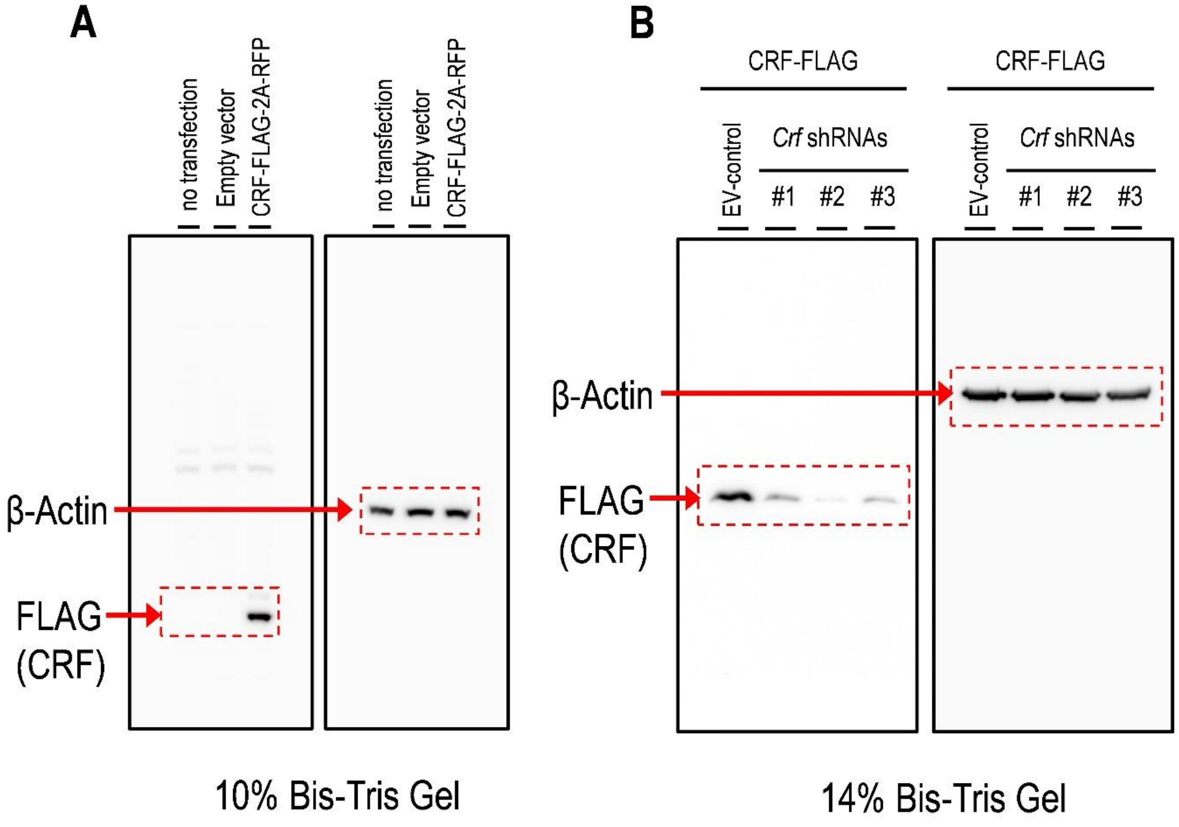

Supplement: Supplementary file 1 [file ijms-23-06286-s001.zip › ijms-1718457-supplementary.pdf]
